# Synthesis, biophysical properties and biological activity of second generation antisense oligonucleotides containing chiral phosphorothioate linkages

**DOI:** 10.1093/nar/gku1115

**Published:** 2014-11-14

**Authors:** W. Brad Wan, Michael T. Migawa, Guillermo Vasquez, Heather M. Murray, Josh G. Nichols, Hans Gaus, Andres Berdeja, Sam Lee, Christopher E. Hart, Walt F. Lima, Eric E. Swayze, Punit P. Seth

**Affiliations:** Isis Pharmaceuticals, Inc., 2855 Gazelle Ct, Carlsbad, CA 92010, USA

## Abstract

Bicyclic oxazaphospholidine monomers were used to prepare a series of phosphorothioate (PS)-modified gapmer antisense oligonucleotides (ASOs) with control of the chirality of each of the PS linkages within the 10-base gap. The stereoselectivity was determined to be 98% for each coupling. The objective of this work was to study how PS chirality influences biophysical and biological properties of the ASO including binding affinity (*T*_m_), nuclease stability, activity *in vitro* and *in vivo*, RNase H activation and cleavage patterns (both human and *E. coli*) in a gapmer context. Compounds that had nine or more Sp-linkages in the gap were found to be poorly active *in vitro*, while compounds with uniform Rp-gaps exhibited activity very similar to that of the stereo-random parent ASOs. Conversely, when tested *in vivo*, the full Rp-gap compound was found to be quickly metabolized resulting in low activity. A total of 31 ASOs were prepared with control of the PS chirally of each linkage within the gap in an attempt to identify favorable Rp/Sp positions. We conclude that a mix of Rp and Sp is required to achieve a balance between good activity and nuclease stability.

## INTRODUCTION

**Figure 1.**
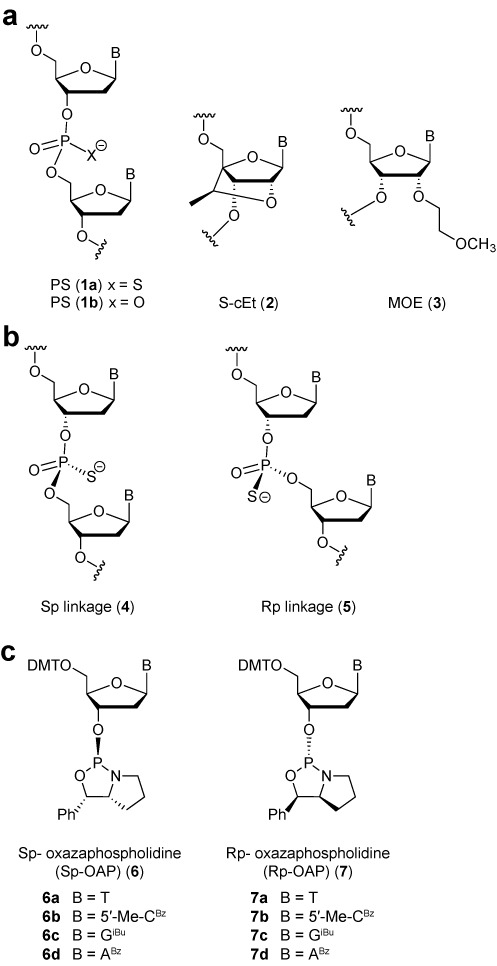
Structures of (**a**) modified oligonucleotides, (**b**) P-chiral Linkages and (**c**) OAP monomers.

The use of chemically modified antisense oligonucleotides (ASOs) as potential therapeutics has received much attention in recent years (1). Chemical modifications are used to enhance a number of ASO drug-like properties such as metabolic stability, RNA-affinity and bioavailability (2). The phosphorothioate (PS) linkage, where one of the non-bridging oxygen atoms of a phosphodiester linkage is replaced with a sulfur atom (see Figure 1a), is one of the most widely investigated nucleic acid chemical modifications in oligonucleotide therapeutics (3). The PS linkage enhances ASO metabolic stability from nuclease-mediated degradation (4). In addition, the PS modification enhances ASO protein binding properties, which facilitates their distribution and internalization into tissues in animals without the aid of complex delivery vehicles or formulations (5).

While the PS modification has been used to enhance drug-like properties of ASOs which function through a variety of antisense mechanisms (6), this modification has been investigated most broadly for ASOs which function through the RNase H mechanism (7). RNase H is a ubiquitously expressed enzyme which selectively degrades the RNA strand of a DNA/RNA heteroduplex (8). Human RNase H1 is comprised of three domains (catalytic, linker and hybrid binding domain (HBD)) and is the key enzyme implicated in the pharmacology of DNA-like ASOs (9). PS DNA is one of very few backbone modifications which improves ASO drug-like properties while maintaining the ability to elicit RNase H enzymatic activity (10), thus making it a privileged modification for this class of ASO therapeutics (3). A peculiar feature of the PS modification is that it exists as a mixture of stereoisomers (designated Sp and Rp, see Figure 1b) since the negative charge resides almost exclusively on the sulfur atom (11). As a result, a uniformly PS modified n-mer oligonucleotide exists as a mixture of 2^(n–1)^ possible stereoisomers.

Previous work, such as Stec's pioneering work in this area (12), has shown that the individual PS stereoisomers possess differential biophysical and biological properties (13–15). For example, the Rp isomer has better *T*_m_ and RNase H activating profile while the Sp isomer shows better nuclease stability (13,14). These results suggested that controlling the chirality of the PS backbone could enhance the overall therapeutic profile of PS DNA ASOs by providing an optimal balance of *T*_m_, nuclease stability and RNase H activity (16–18). However, much of the work was carried out using first generation ASO designs comprised only of PS DNA ASOs (12,17,19). This design is sub-optimal for therapeutic applications and has been largely supplanted with second generation ‘gapmer’ ASOs which are more potent and highly stabilized from exonuclease-mediated degradation.

Second generation ASOs are chimeric oligonucleotides with a central gap region of 8–16 PS DNA nucleotides flanked on either end with 2–5 affinity-enhancing 2′-O-alkylated RNA monomers. One such modification is 2′-O methoxyethyl, or MOE (**3**, Figure 1a) (20). Kynamro, a fully PS modified second generation ASO (21), was recently approved by the FDA for the treatment of familial hypercholesterolemia (22). In addition, there are over 30 RNase H active ASOs in active development for the treatment of a variety of diseases such as cancer, metabolic, cardiovascular and genetic diseases. Further advances in ASO design have yielded next generation designs where the MOE nucleotides can be replaced with other classes of affinity-enhancing nucleotides (23–25) such as 2′,4′-constrained ethyl nucleotides or cEt (**2**, Figure 1a).

In this study, we sought to investigate if controlling chirality of the PS linkage in the DNA gap region can modulate the therapeutic profile of next generation gapmer ASOs. This choice was based on the crystal structure of human RNase H1 which showed that the catalytic domain makes contacts with three phosphodiester linkages in the DNA backbone (26) while the HBD contacts the bottom face of the DNA sugars (27). In the context of gapmer ASOs, it was previously shown that the HBD most likely binds in the vicinity of the 2′-modified nucleotides in the flanks while the catalytic domain binds in the gap region of gapmer ASOs (28,29). In this study, we found that controlling PS chirality in the gap region of second generation ASOs provides no significant benefits for therapeutic applications relative to the mixture of stereo-random PS ASOs. Furthermore, the added complexity and cost associated with the synthesis and characterization of chiral PS ASOs minimizes their utility especially for RNase H active gapmer ASOs.

## MATERIALS AND METHODS

### Oxazaphospholidine (OAP) monomer synthesis

Oxazaphospholidine (OAP) monomers **6a**–**d** and **7a**–**d** (Figure 1c) were prepared in a manner similar to that described by Wada *et al.* (30). The procedures were modified in order to minimize chromatography and to make the process more amenable to scale up. For details see Supplementary Data, Figures S1 and S2.

### Oligonucleotide (ON) synthesis

ONs were prepared on an ABI 394 DNA/RNA synthesizer (2 μmol scale) or an AKTA oligopilot 10 (40 μmol scale) using polystyrene-based NittoPhase unylinker^TM^ support (315 μmol/g). Fully protected nucleoside phosphoramidites were incorporated using standard solid-phase oligonucleotide synthesis conditions: i.e. 3% dichloroacetic acid in dichloromethane (DCM) for deblocking, 1 M 4,5-dicyanoimidazole with 0.1 M *N*-methylimidazole in acetonitrile as activator, 10% acetic anhydride in THF and 10% *N*-methylimidazole in THF/pyridine for capping and 0.2 M phenylacetyl disulfide in pyridine:acetonitrile 1:1 (v:v) for thiolation. DNA amidites were prepared at 0.1 M in acetonitrile:toluene 1:1 (v:v) and were incorporated using 5 min coupling time. OAP monomers were prepared at 0.2 M in acetonitrile:toluene 1:1 (v:v) and were coupled utilizing two applications of OAP, with a 6 min contact time for each pass. After conclusion of the synthesis, the 5′-dimethoxytrityl group (DMT) group was removed. The solid support was suspended in aqueous ammonia (28–30 wt.%) and heated at 55°C for 48 h. The reaction mixture was allowed to come to room temperature, and the solid support was filtered and washed with a solution of EtOH and H_2_O 1:1 (v:v). The washings and filtrate were combined and were concentrated under reduced pressure to ∼ 20% of the original volume. The residue obtained was dissolved in water and purified by high performance liquid chromatography (HPLC) on a strong anion exchange column (Mono Q, GE Healthcare, 16/10, 20 ml, 10 mm, ionic capacity 0.27–0.37 mmol/ml, A = 100 mM ammonium acetate, 30% aqueous acetonitrile, B = 1.5 M NaBr in A, gradient of 20–50% B over 40 min, Flow 2.0 ml/min, λ = 260 nm). Oligonucleotides were desalted over HiPrepTM 26/10 columns (GE Healthcare) (40 μmol scale) or by utilization of C18 reverse-phase cartridges (2 μmol scale). The oligonucleotides were lyophilized to give an overall isolated yield of 6–8% based on the initial loading of the solid support.

### Oligonucleotide analysis and determination of stereospecificity

Oligonucleotides were characterized by ion-pair-HPLC coupled mass spectrometry (MS) analysis with Agilent 1100 MSD system. PS oligonucleotides that terminate at the 5′-end in a 4, 4′-DMT are resolved chromatographically into two peaks on the basis of the absolute configuration of the internucleotide linkage located at the 5′-end of the oligonucleotide. The first peak corresponds to the group of diastereoisomers with *S*p configuration at the 5′-most internucleotide linkage and the second peak is the group of diastereoisomers Rp configuration at the 5′-most internucleotide linkage (*vide infra*). Comparison of the relative ultraviolet (UV) areas of the two peaks is a direct measure of Sp/Rp ratio at the 5′-most internucleotide linkage.

### Thermal denaturation studies (*T*_m_)

ASO and RNA were mixed in 1:1 ratio (4 μM duplex) in buffer containing 10 mM phosphate, 100 mM NaCl and 10 mM ethylenediaminetetraaceticacid (EDTA) at pH 7.0. The duplex was denatured at 85°C and slowly cooled to the starting temperature of the experiment (15°C). Thermal denaturation temperatures (*T*_m_ values) were measured in quartz cuvettes (pathlength 1.0 cm) on a Cary 100 UV/visible spectrophotometer equipped with a Peltier temperature controller. Absorbance at 260 nm was measured as a function of temperature using a temperature ramp of 0.5°C/min. *T*_m_ values were determined using the hyperchromicity method incorporated into the instrument software.

### Cell preparation and evaluation of ASO activity *in vitro*

Liver perfusion. Mouse liver was perfused as previously described with minor modifications (31–33). Briefly, mice were anesthetized with an intraperitoneal injection of ketamine/xylazine. Inferior vena cava was catheterized and clamped. The liver was perfused with Hank's Balanced Salt Solution (Life Technologies) and mesenteric vessel was cut for drainage. The liver was subsequently perfused with collagenase (Roche). Following the perfusion, the liver was removed and gently massaged through sterile nylon mesh. Cells were washed in Williams E (Life Technologies) containing 10% fetal calf serum, 4-(2-hydroxyethyl)-1-piperazineethanesulfonic acid (HEPES), L-glutamine and antibiotic/antimycotic. Parenchymal cells were separated from non-parenchymal via centrifugation.

Electroporation. 10× ASO was diluted to 1× by either hepatocytes or b.END cells at a density of 35,000 cells/well for hepatocyte cells or 20,000 cell/well for b.END cells. Hepatocyte cells were electroporated at 165 V and b.END cells at 115 V for a pulse length of 6 ms. Cell/oligo mixture was transferred into a 96-well tissue culture plate containing Williams E medium (Life Technologies) for hepatocytes or Dulbecco's modified Eagle's medium (Life Technologies) for b.END and 10% fetal bovine serum. Cells were incubated overnight at 37°C in 10% CO_2_. Cells were lysed 16 h post transfection and total RNA was isolated and purified using RNeasy 3000 Bio Robot (Qiagen). Reduction of target mRNA was determined by quantitative reversetranscriptase-polymerase chain reaction (qRT-PCR) as previously described (34). The primer-probe sequences used for detection of SRB were forward TGACAACGACACCGTGTCCT, reverse ATGCGACTTGTCAGGCTGG and probe CGTGGAGAACCGCAGCCTCCATT. Primer-probe sequences for androgen receptor (AR) detection were forward TTAACGTCCTGGAAGCCATTG, reverse CAAAGGAATCTGGTTGGTTGTTG and probe CCAGGAGTGGTGTGTGCCGGACAT. Target mRNA levels were normalized to total RNA using RiboGreen (Life Technologies). IC_50_ curves and values were generated using Prism 4 software (GraphPad).

### Protocols for animal experiments

Animal experiments were conducted according to American Association for the Accreditation of Laboratory Animal Care guidelines and were approved by the Animal Welfare Committee (Cold Spring Harbor Laboratory's Institutional Animal Care and Use Committee guidelines). Male Balb/c mice (Charles River Laboratories), aged 6–8 weeks and *n* = 3, were maintained at a constant temperature of 23°C and were allowed standard lab diet and water. Dosing solutions were prepared in phosphate-buffered saline, sterile filtered and quantified. Mice were dosed, at the indicated dosage, twice a week for two weeks by subcutaneous injection. Mice were sacrificed 48 h after the last injection. Animals were anesthetized with isoflurane and terminal bleed was performed as previously described (35). Immediately following terminal blood draw, mice were sacrificed by cervical dislocation while under anesthesia. Plasma chemistries (alanine amino transferase, aspartate amino transferase, blood urea nitrogen (BUN), and total bilirubin) were measured on an Olympus AU480 Clinical Analyzer (Beckman Coulter). Liver, kidney and spleen weights were taken and liver tissue was homogenized in guanidine isothiocyanate (Life Technologies) containing 8% β-mercaptoethanol (Sigma) immediately following the sacrifice. Liver homogenate was loaded onto Purelink PCR columns (Life Technologies) and total RNA was purified according to manufacture instructions. Reduction of target mRNA expression was determined by qRT-PCR as described above. Target mRNA levels were normalized to total RNA as determined by RiboGreen.

### Determination of liver concentrations of nucleic acid agents using LC-MS

cEt gapmer ASOs were extracted from liver samples along with five to eight standards of each analyte prepared in control liver and analyzed by LC-MS using previously described methods (36,37). Briefly, a 27-mer MOE/DNA PS oligonucleotide was added as an internal standard to each sample and analyte standard before extraction with phenol/chloroform and then solid-phase extraction of the resulting aqueous extract using phenyl-functionalized silica sorbent (Biotage). Mass measurements were made using a single quadrupole mass spectrometer and selected ion monitoring of ion *m*/*z* corresponding to the −3 charge state of cEt gapmer and −5 charge state of the internal standard. Peak areas from extracted ion chromatograms were determined for each analyte and normalized to the peak area of the internal standard. Concentrations were determined using a trend line established with the extracted standards and reported as μg/g liver.

### RNase H cleavage and kinetics

RNA was 5′-end labeled with ^32^P using 20 U of T4 polynucleotide kinase, 120 pmol (7000 Ci/mmol) of [γ-^32^P]ATP, 40 pmol RNA, 70mM Tris–HCl, 10 mM MgCl_2_ and 50 mM dithiothreitol, at pH 7.6. The labeling reaction was incubated at 37°C for 30 min followed by heating at 90°C for 1 min. Labeled RNA was purified using 12% denaturing polyacrylamide gel. ASO (200 nM), unlabeled 19-mer RNA (100 nM) and a small amount of ^32^P-labeled RNA was mixed in hybridization buffer [20 mM Tris, 20 mM KCl (pH 7.5)] and heated to 90°C for 2 min. To the hybridization mixture was added 0.1 mM TCEP, 1mM MgCl_2_ and 40 U of RNaseOUT and incubated at 37°C for 1 h. The human RNase H1 enzyme was incubated in dilution buffer [20mM Tris, 50 mM KCl and 2 mM tris(2-carboxyethyl)phosphine (TCEP) (pH 7.5)] for 1 h at rt. Enzyme solution (1/25 ml duplex solution) was added to duplex solution and incubated at 37°C. At time point's aliquots were removed and reaction quenched in loading buffer and snap-frozen on dry ice. Cleavage products were separated by 12% denaturing polyacrylamide gel and products were quantitated on a Phosphor-Imager. We have found that the absolute initial rates vary as a function of the RNaseH assay preparation; however, the relative rank ordering between experiments remains consistent.

## RESULTS

### Synthesis of chiral PS ASOs

The various methods that can be used to synthesize P-chiral PS linkages has been recently reviewed (18,38). The use of bicyclic OAP monomers described by Wada *et al.* (30) (Figure 1c) was selected for the following reasons: (i) stereoselectivity was reported to be > 99%, (ii) reaction conditions were mild, (iii) no tedious chromatography was required and (iv) the method was compatible with automated oligonucleotide synthesizers with minimal modifications. Using this approach, not only is it possible to study the properties of PS-gapmer ASOs that have stereo-regular gaps, but we can also prepare discrete Rp/Sp gap configurations in an attempt to more accurately correlate activity with PS chirality. Two target sequences were selected for this study. The first was a previously identified potent 3–10–3 cEt gapmer (**A5**) targeting scavenger receptor B1 (SR-B1), a ubiquitously expressed gene whose physiological role is related to cholesterol uptake into tissues (39,40). The SR-B1 receptor has also been implicated as an entry point for viruses such as hepatitis C virus and other pathogens (41) and its down-regulation could provide a therapeutic benefit by preventing entry of infectious pathogens into host cells. The second ASO sequence that we studied (**A10**) was designed to inhibit the production of the AR—a well-validated target associated with prostate cancer (42).

### Evaluation of OAP monomer coupling and method development

**Figure 2.**
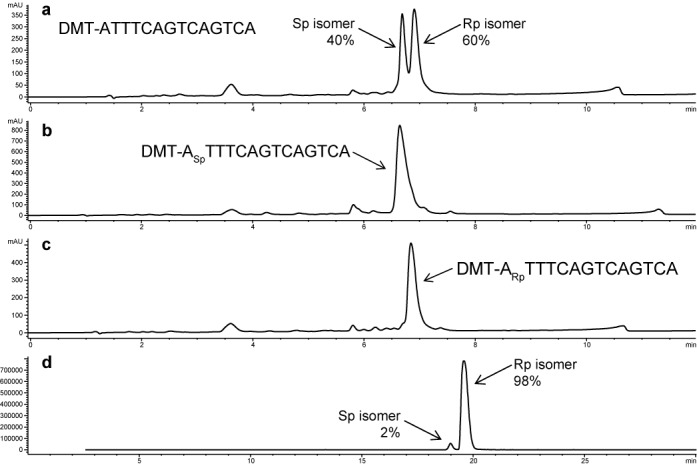
Evaluation of the stereoselectivity of OAP couplings. Each nucleoside followed by a subscript ‘Sp’ indicates a 3′-Sp linkage and each nucleoside followed by a subscript ‘Rp’ indicates a 3′-Rp linkage. All other linkages are stereo-random PS. (**a**) 15-mer ASO with a stereo-random PS 5′-terminal linkage. HPLC (UV area suggests a mixture of stereoisomers with a ratio of 40:60 Sp:Rp; (**b**) 15-mer ASO with a 5′-Sp terminal linkage (HPLC, UV); (**c**) 15-mer with a 5′-Rp terminal linkage (HPLC, UV); (**d**) analysis of 15-mer (5′-Rp terminal linkage) by extracted ion chromatogram mass spectroscopy (EIC-MS) shows that OAP couplings are typically 98% selective.

To evaluate the coupling efficiency and stereoselectivity of the reported OAP method a series of 15-mers was prepared. The first 14 bases were synthesized using standard phosphoramidites and standard conditions (i.e. the first 14 bases were 2′-deoxynucleosides and the first 13 PS linkages were stereo-random), followed by a 5′-terminal nucleotide which was connected by either a diastereomeric PS linkage, an Sp linkage or an Rp linkage. The resulting 15-mers were analyzed by RP-HPLC (UV area) as described in the Materials and Methods section. The chromatograms can be found in Figure 2 and show that the Rp- and Sp-isomers can be resolved. Note that the addition of OAP monomers involves compete inversion of stereochemistry; i.e. addition of an Sp OAP (Figure 1, **6**) results in an Rp linkage (Figure 1, **5**), while addition of an Rp OAP (Figure 1, **7**) results in an Sp linkage (Figure 1, **4**) Figure 2a shows a 15-mer with a terminal base that was coupled using a standard phosphoramidite chemistry (i.e. stereo-random PS linkage). Based on UV area, the ratio of Sp to Rp (faster-eluting peak to slower eluting peak, respectively) was 40:60, which is consistent with previous reports (43). Figure 2b shows the HPLC of a 15-mer with a 5′-terminal Sp linkage and indicated that the coupling occurred with high stereoselectivity. Similarly, analysis of the 5′-terminal Rp ASO by HPLC also showed that the use of OAP monomers results in couplings that are highly stereoselective (Figure 2c). In order to determine the stereoselectivity of the OAP monomers more precisely, the products were also analyzed by extracted ion chromatography (EIC) (Figure 2d). Using this method, we were able to determine that 98% stereoselectivity was typical for each of the OAP monomers (see also Supplementary Data, Figure S3)

OAP monomers do not couple as efficiently as phosphoramidite monomers. A variety of conditions were tested in an attempt to improve coupling efficiency and to push the coupling reactions to completion. Choice of activators, thiolating agents and/or capping reagents did not increase the coupling efficiency, though it is interesting to note that the stereoselectivity was unaffected by these variables (Supplementary Data, Figure S4). By increasing the coupling time, concentration and use of multiple applications of monomer, we were able to achieve ∼ 93% coupling efficiency per incorporation. Oligomers containing phosphodiester linkages were observed as major by-products either due to incomplete manual sulfurization or oxidation of the PS bonds post-synthesis. Isolated yields of 6–8% were typical after ASO purification.

### Thermal stability measurements of uniform-gap ASOs

A set of four ASOs was prepared for each sequence that was tested: (i) full length PO, (ii) full length PS (stereo-random), (iii) uniform Rp-gap and (iv) uniform Sp-gap. ASOs **A1**–**A8** were designed to target mRNA for the SR-B1 receptor and ASOs **A9**–**A16** were designed to target mRNA for the AR (see Table 1 ASOs were hybridized with complementary mRNA and were tested for affinity in a UV thermal denaturing study to acquire thermal melting curves and assess the temperature of melting (*T*_m_) (Table 1).

**Table 1.**
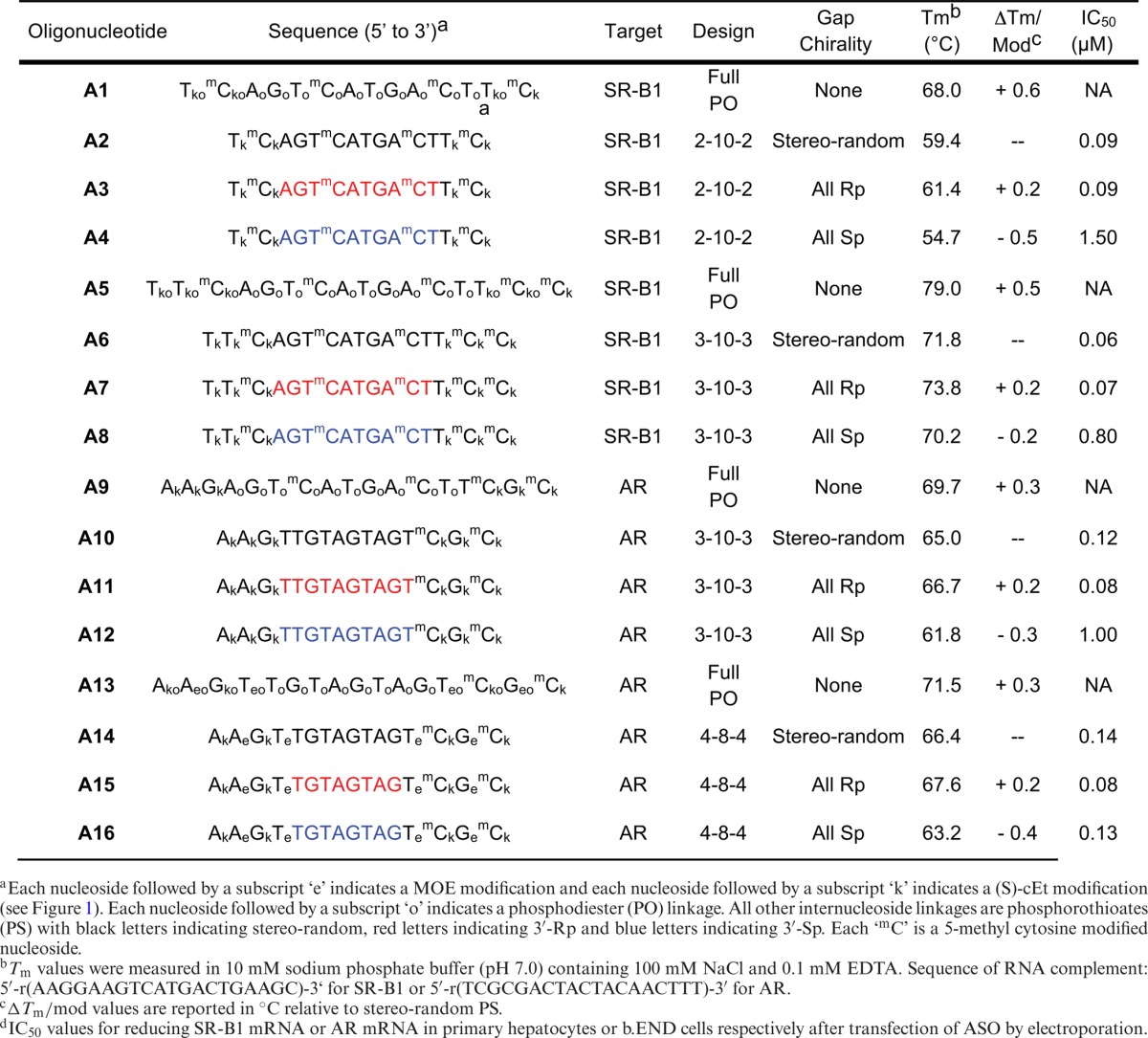
*In vitro* activity and *T*_m_ data exhibited by P-chiral gapmer ASOs relative to stereo-random PS PS and PO

For each set of ASOs, the following observations held true: (i) Stereo-random PS ASOs (**A2**, **A6**, **A10** and **A14**) were found to be less thermally stable than their corresponding full PO ASOs (**A1**, **A5**, **A9** and **A13**, respectively) by ∼ 0.5°C per linkage, which is consistent with previous studies; (ii) The incorporation of Rp PS linkages in the gap (**A3**, **A7**, **A11** and **A15**) led to an increase in *T*_m_ by ∼ 0.2°C per incorporation relative to the *T*_m_ of stereo-random PS ASOs (**A2**, **A6**, **A10** and **A14**, respectively; (iii) The incorporation of Sp PS linkages in the gap (**A4**, **A8**, **A12** and **A16**) led to a decrease in *T*_m_ by ∼ 0.4°C per incorporation relative to the *T*_m_ of stereo-random PS ASOs (**A2**, **A6**, **A10** and **A14**, respectively). The net result is the following rank order for thermal stability: PO > Rp > stereo-random PS > Sp. This trend is conserved regardless of sequence, ASO length or gap size.

To further explore the effect that multiple chiral PS linkages has on *T*_m_, a series of oligonucleotides with P-chiral linkages was prepared based on **A17**, an ASO with well-documented thermal melting properties (see Table 2) (44). The following observations seemed to hold true: (i) Increasing Rp or Sp content led to a non-linear decrease in *T*_m_ relative to that of the parent ASO (**A17**); (ii) Incorporation of Sp linkages has a larger negative affect on *T*_m_ than incorporation of Rp linkages; and (iii) The ASOs containing a mixture of Rp- and Sp-linkages lies in between that of pure Rp and Sp gaps.

### S1 endonuclease selectively degrades all-Sp gap ASOs

ASOs **A10**–**A12** (10-base gap AR ASO) and **A14**–**A16** (8-base gap AR ASOs) were tested for stability against S1 endonuclease to confirm configurational assignment. ASOs were incubated with nuclease S1 from *Aspergillus oryzae* (Sp-specific) at room temperature over a time course. ASOs and digestion products were analyzed by LCMS. As expected, the digestion profile showed that the all-Sp gap compounds **A12** and **A16** were rapidly digested (< 10 min) while the all-Rp gap compounds **A11** and **A15** were found to be significantly more stable than the Sp-gap ASOs (**A12** and **A16**) and the diastereomeric PS parent ASOs (**A10** and **A14**) (see Supplementary Data, Figures S5–S7).

### RNase H activity and initial rates of cleavage

ASOs were prepared at 200 μM concentration and were incubated for 1 h at 37°C with complementary mRNA that had been 5′-end labeled with ^32^P. The resulting duplex was then incubated with *Escherichia coli* (*E. coli*) RNase H (20-fold excess) or human RNase H1 (50 fold excess) at 37°C. Aliquots were removed over time and cleavage products were separated by 12% denaturing polyacrylamide gel, and quantitated on a Phosphor-Imager.

**Figure 3.**
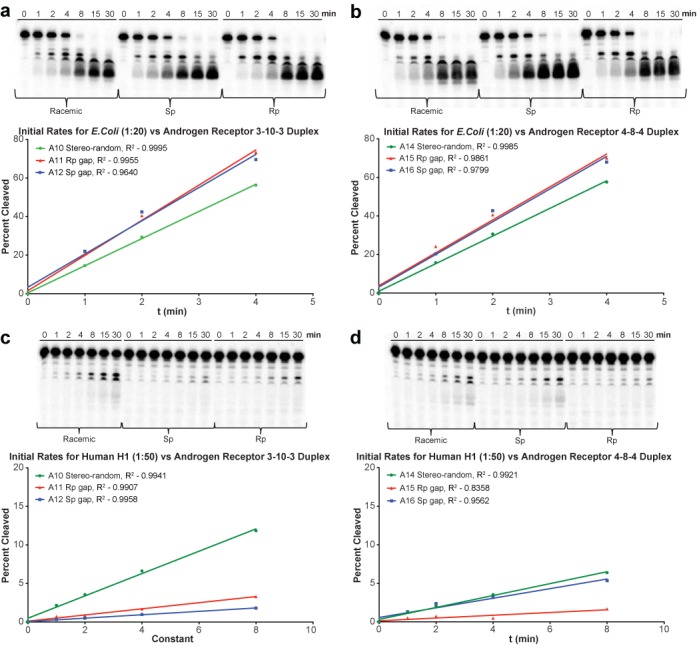
RNase H1 cleavage of P-32 labeled mRNA with AR ASO/RNA duplexes. For each set of lanes, the incubation time was 0, 1, 2, 4, 8, 15 and 30 min. (**a**) *E. coli* RNase H1 and 10-base gap ASO/RNA duplex; (**b**) *E. coli* RNase H1 and 8-base gap ASO/RNA duplex; (**c**) human RNase H1 and 10-base gap ASO/RNA duplex; (**d**) human RNase H1 and 8-base gap ASO/RNA duplex.

Based on the initial rates of cleavage using RNase H1 derived from *E. coli*, the results in Figure 3 indicate that the all-Rp gap AR ASOs (**A11** and **A15**) and the all-Sp gap AR ASOs (**A12** and **A16**) were more active than their corresponding stereo-random PS parent ASOs (**A10** and **A14**, respectively). This observation held true for both the 10-base compounds (Figure 3a) as well as the 8-base gap compounds (Figure 3b). Although it has been reported that the Rp isomer is a better activator of bacterial RNase H1 (13,17), we found that the difference in initial rates of cleavage for the all-Rp gap ASOs versus the all-Sp gap ASOs was minimal.

The results in Figure 3c and d show that human RNase H1 behaves differently than bacterial RNase H1. Human RNase H1 has both a binding domain and a catalytic domain, whereas bacterial RNase H has a catalytic domain only (10,45). As a result, human RNase H has a more specific binding requirement and thus exhibits activity and cleavage that is less prolific than *E. coli* RNase H1. In the case of human RNase H1, the all-Rp gap AR ASOs (**A11** and **A15**) and all-Sp gap AR ASOs (**A12** and **A16**) were less active than their corresponding stereo-random PS parent ASOs (**A10** and **A14**, respectively). In the case of the 10-base gap compounds there was very little difference in initial rates of cleavage between the all-Rp and the all-Sp gap compounds (**A11** and **A12** respectively, Figure 3c). In the case of the 8-base gap ASOs, however, the Sp-gap isomer (**A16**) was similar to the stereo-random parent, which were both more active than the Rp-gap isomer (**A15**).

### Evaluation of uniform Sp- and Rp-gap ASOs in cell culture

**Figure 4.**
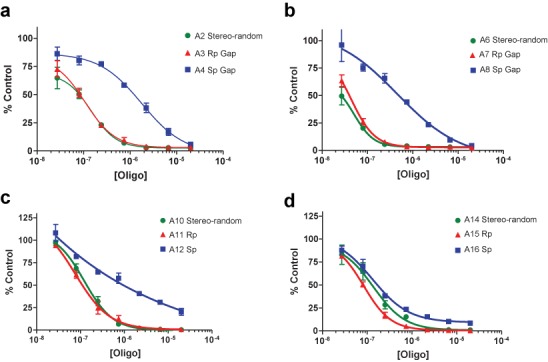
P-Chiral Gapmer ASO dose response curves. ASOs were transfected into cells via electroporation. mRNA reduction was normalized to RiboGreen. (**a**) Reduction of SR-B1 mRNA in hepatocytes, 2-10-2 ASO motif; (**b**) reduction of SR-B1 mRNA in hepatocytes, 3-10-3 ASO motif; (**c**) reduction of AR mRNA in b. END cells, 3-10-3 ASO motif; (**d**) reduction of AR mRNA in b.END cells, 4-8-4 ASO motif.

Uniform gap ASOs (**A2**–**A4**, **A6**–**A8**, **A10**–**A12** and **A14–16**) were tested for activity in cell culture in a seven-point dose response assay. ASOs were transfected into mouse primary hepatocytes (SR-B1 ASOs) or b.END cells (AR ASOs) at 20, 6.67, 2.22, 0.74, 0.25, 0.08 and 0.03 μM via electroporation. The cells were incubated overnight and were lysed 16 h post treatment for RNA purification and analysis (Figure 4). The IC_50_ values were calculated and can be found in Table 1. The 10-base all-Rp gap compounds (**A3**, **A7** and **A11**) exhibited activity that was virtually identical to that of the corresponding diastereomeric PS parent ASOs (**A2**, **A6** and **A10**, respectively). Conversely, the 10-base all-Sp gap compounds (**A4**, **A8** and **A12**) were significantly less active. The exception to the trend was the 8-base all-Sp and all-Rp gap AR ASOs (**A15** and **A16**), both of which exhibited activity *in vitro* that was very similar to that of the stereo-random parent (**A14**).

### Evaluation of ASOs A6–A8 in animal experiments

**Figure 5.**
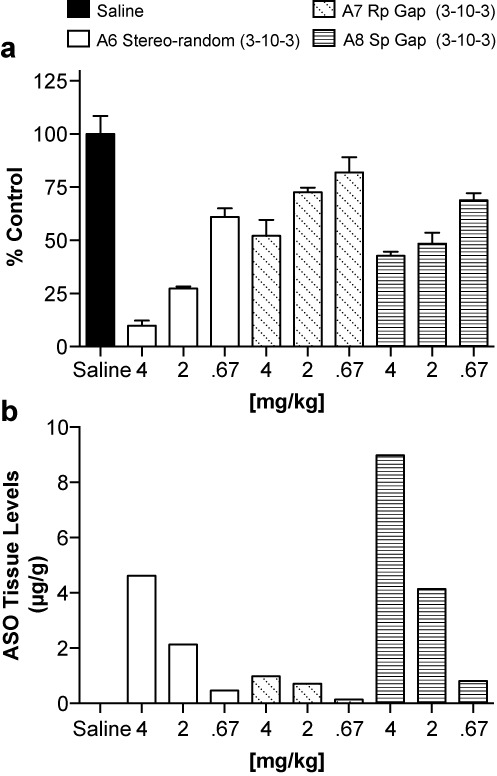
Evaluation of P-chiral gapmers *in vitro*. (**a**) Reduction of SR-B1 mRNA in liver tissue (normalized to RiboGreen); (**b**) amount of intact ASO recovered from liver tissue.

SR-B1 ASOs **A7** (all-Rp gap) and **A8** (all Sp gap) were selected for evaluation in animal experiments and were compared to the performance of the stereo-random parent ASO **A6**. Mice (*n* = 3 per group) were injected subcutaneously with 0.67, 2.0 and 4.0 mg/kg of SRB-1 ASOs **A6**, **A7** and **A8** twice a week for 2 weeks. The animals were sacrificed 48 h after the last ASO dose and the SRB-1 mRNA in liver was measured by quantitative RT–PCR. Target mRNA was normalized to total RNA levels via RiboGreen (see Figure 5a).

The data in Figure 5a show that both chirally controlled compounds **A7** and **A8** were similar in activity, and that both compounds were less active than the stereo-random parent compound **A6**. The amount of intact ASO remaining in liver tissue was quantified and is shown in Figure 5b. Consistent with our previous studies of PS-modified gapmer ASOs, drug accumulation in liver was dose-dependent (40). Although the level of Sp-gap ASO **A8** found in the tissue was high relative to **A7** and **A6**, we know from the *in vitro* activity assay that the 10-base Sp-gap ASOs exhibit poor activity relative to Rp-gap and stereo-random PS parent ASO. Conversely, Rp-gap ASOs exhibited activity similar to that of diastereomeric PS ASOs *in vitro*, but levels of Rp-gap ASO **A7** found in the tissue were low relative to **A6** and **A8**.

### Testing of additional panel of nucleases

The relatively low abundance of the Rp-gap ASO recovered in tissue suggests that the yet unidentified nuclease(s) that metabolize gapmer ASOs have a preference for the Rp isomer. We have shown that Sp-gap ASOs are more susceptible to degradation by S1 endonuclease while Rp-gap ASOs are more stable (Supplementary Data, Figures S5–S7). There are multiple reports in the literature that indicate that the Rp PS linkages are rapidly metabolized by exonucleases such as snake venom phosphodiesterase (svPDE), however, gapmer ASOs are resistant to exonuclease degradation as reported in the literature (30,46–48) and confirmed in this body of work (see Supplementary Data, Figure S8). Additionally, ASOs **A6**, **A7** and **A8** were tested against a panel of nucleases including micrococcal endonuclease, human APE-I, porcine DNase II, svPDE and DNA polymerase 1 (49–52). We also tested these ASOs in mouse serum as well as mouse hepatocyte homogenate. We found that micrococcal endonuclease from *Staphylococcus aureus* was specific for the Rp-gap ASO (**A7**) with the Sp-gap ASO (**A8**) being significantly more stable (see Supplementary Data, Figure S8) (53). In all other cases, we found that all three ASOs were stable over the time of the assay (*t* $\frac{1}{2}$ >> 24 h), and there was no significant difference between the all-Sp gap and the all-Rp gap compounds.

### Statistical approach to the identification of favorable Rp/Sp gap configurations

The incorporation of Rp linkages increases the affinity of ASOs to their target RNA, however ASOs with long stretches of sequential Rp linkages (10 or more) are susceptible to degradation by exonucleases *in vivo*. Conversely, ASOs that contain long stretches of Sp linkages were found to be more stable to exonucleases but gave rise to compounds with lower target affinity and lower activity *in vitro*. Thus, it appears likely that a mixture of Rp and Sp is necessary to achieve a balance between good target affinity, activity and nuclease stability. The stereospecific OAP coupling method will enable us to synthesize discrete Sp/Rp gap configurations in an attempt to determine whether or not specific combinations of Sp and Rp result in ASOs that were significantly more potent (5×) than the parent ASO prepared under stereo-random conditions (i.e. ‘super isomers’).

SR-B1 ASO **A6** was selected as the parent ASO for this study. Twenty-nine isomers (**A27**–**A55**) were synthesized utilizing the bicyclic OAP monomers to control the chirality of each PS linkage within the gap. These isomers were selected from a master list of the 1024 possible isomers without bias or preconceived notion of performance based on the Rp/Sp gap configuration. The ASOs were tested for activity in cell culture in a five-point dose response assay and IC_50_ values were calculated in an attempt to identify trends and patterns that correlate Rp/Sp pattern/configuration with activity (Table 3). Mass spectrometry data for all ASOs in this report can be found in Supplementary Data, Figure S9. Consistent with previous results, the 10-base Rp gap ASO (**A7**) exhibited activity that was equal to that of the stereo-random PS parent ASO **A6**, while the activity of the 10-base Sp gap ASO (**A8**) was greatly decreased. It is interesting that **A28**, which has nine sequential Sp linkages, exhibits moderate activity. Although there is not a compound with eight sequential Sp-linkages in this sample set, there are two compounds that have a total of eight Sp linkages broken up with Rp linkages (**A34** and **A36**), both of which have similar activity to the diastereomeric PS parent. This suggests that 9–10 sequential Sp linkages are detrimental to activity, but eight or less is tolerated. This point can be further illustrated by comparing the activity AR ASOs **A12** and **A16** (Table 1): the 10-base Sp AR ASO (**A12**) was found to be inactive while the 8-base Sp AR ASO (**A16**) was nearly as active as the stereo-random PS 8-base Gap AR ASO (**A14**). With the exception of **A8** and **A28**, the remainder of the compounds in Table 3 were found to exhibit activity within 2× of that of the diastereomeric PS parent **A6**.

**Table 2.**
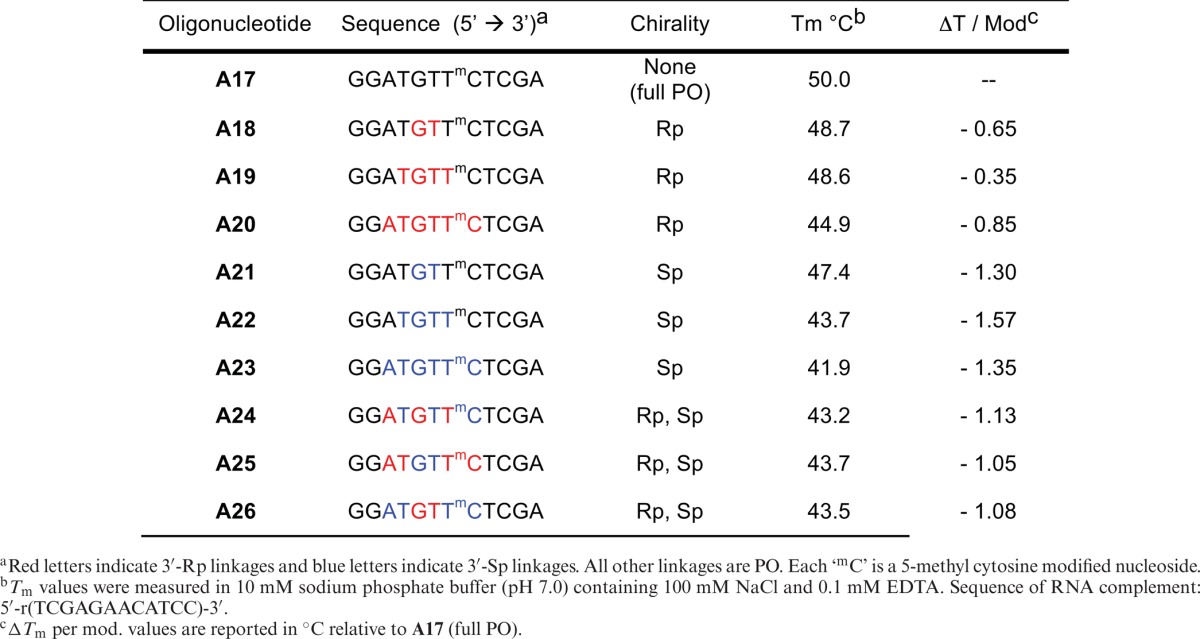
Evaluation of the contribution of multiple Rp- and Sp-linkages to thermal stability

**Table 3.**
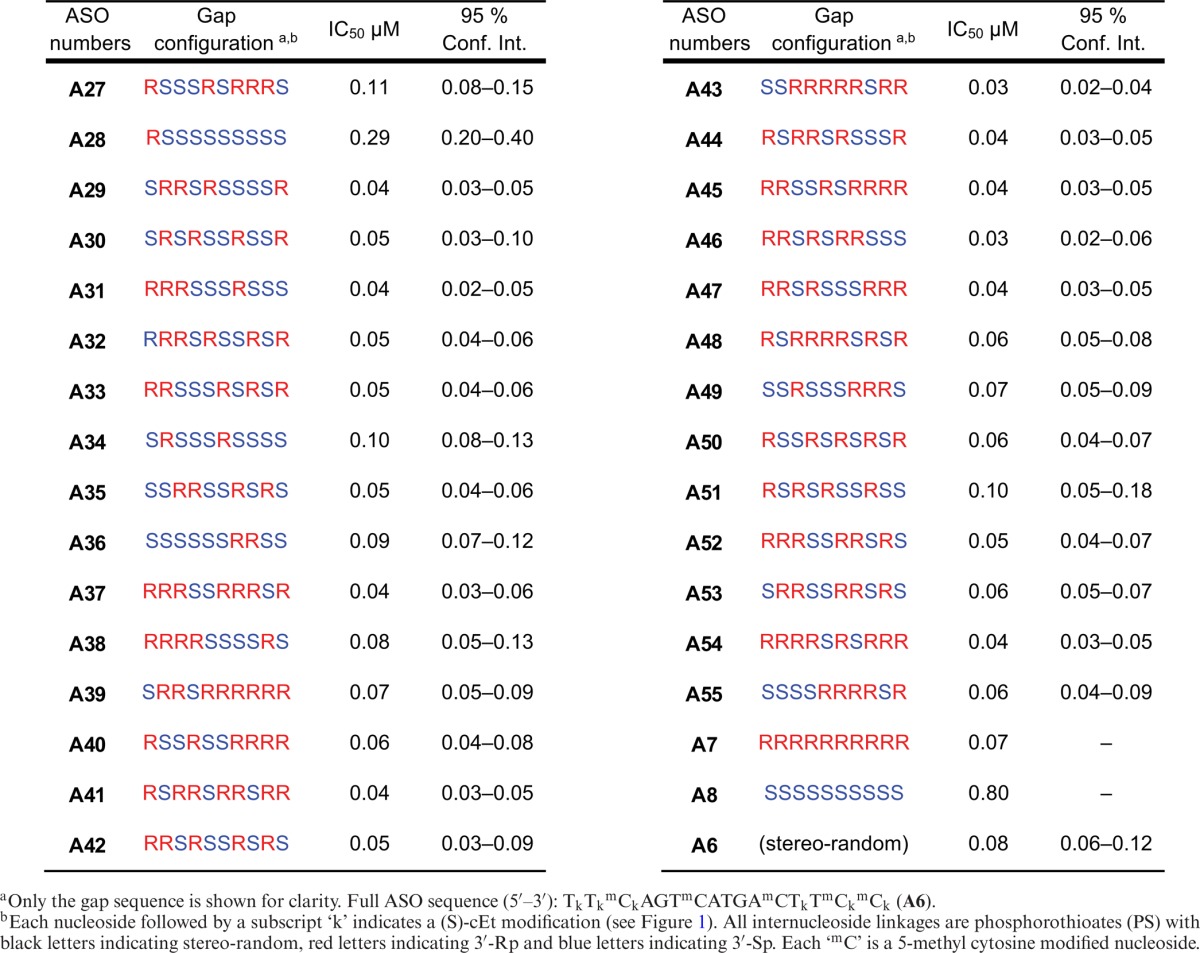
Gap configuration and IC_50_ values of P-chiral gapmer ASOs

## DISCUSSION

We have found the recent developments of Wada *et al.* (30) using bicyclic OAP monomers to be a practical method for the synthesis of chirally pure PS linkages of ASO PS, albeit in a lower yield than traditional phosphoramidites chemistry. Using this methodology, we were able to prepare ASOs and study the effects of chirally pure Rp and Sp PS gaps for the first time in a ‘gapmer’ context. Previous studies (12–15,17–19) have been carried out on chirally pure ASOs using first generation chemistry (i.e. contiguous 2′-deoxynucleoside phosphoramidites) without a MOE, cEt or other second generation stabilized ‘wings’. Without this stabilization, the Rp isomers were metabolized by exonucleases much faster than the Sp isomer. The ‘gapmer’ ASO design circumvents these issues associated with first generation ASO chemistry.

In comparison to the first generation studies reported in the literature, the trends in duplex thermal stability of the gapmer ASOs are similar. As shown in Table 1, the PO isomers exhibit higher affinity towards complementary RNA than their corresponding stereo-random PS ASOs. The Rp isomers have a stabilizing effect relative to the stereo-random PS while the Sp isomers have a destabilizing effect. Interestingly, as shown in Table 2, multiple incorporations of either Rp or Sp isomers, in a PO context, results in non-linear decreases in *T*_m_, with the Rp isomer offering less of a decrease than the Sp isomer, suggesting that all-Sp ASOs are a lower-limiting case in thermal stability.

The decreased thermal affinity of the Sp isomer seems to correlate with the *in vitro* activity. For example, when comparing the difference in IC_50_ with the all-Sp or all-Rp gapmers (Table 1), a substantial decrease in activity was seen with the all-Sp isomer compared to the all-Rp or stereo-random isomer. Interestingly, when one or two Rp isomers are inserted back into the all-Sp context (compare: **A8**, **A28**, **A36** and **A34** in Table 3.) the activity is restored (or nearly restored) to that of the stereo-random parent. This parallels the *T*_m_ studies in that the all-Sp seems to be a limiting case, where runs of 9–10 Sp isomers in the gap significantly decrease cell culture activity. It is noteworthy that we were unable to find an isomer even two times more active (within the 95% confidence interval) from the stereo-random parent. This suggests that, at least in terms of *in vitro* activity, most of the isomers are nearly equivalent in activity except for certain limiting cases of all-Sp or at least runs of 9-Sp isomers which were less active. While it is certainly possible that among the 1024 potential isomers exists within a set of ‘super isomer(s)’ (i.e. with >5× activity *in vitro*), the fact that we chose our specific absorption rate set randomly suggests that this is unlikely. Unfortunately, a herculean, exhaustive screen would be required to answer this question definitively. For further discussion regarding the probability of finding a ‘super-isomer’, see Supplementary Data, Figure S10.

There does not appear to be a correlation of the RNase H activity to either the *T*_m_ or cell culture data of our 3-10-3 motif. While human RNase H1 has both a binding domain and a catalytic domain, the bacterial RNase H has a catalytic domain only (10,45). As a result, human RNase H has a more specific binding requirement and thus exhibits activity and cleavage that is less prolific than *E. coli* RNase H1. The fact that the Rp isomer (**A11**), Sp isomer (**A12**) and stereo-random parent (**A10**) all have initial rates that are comparable for the *E. coli* version, but both the chirally-pure ASOs are significantly less active than the stereo-random isomer is probably reflective of this additional selectivity. It appears, however, that RNase H initial rates are not rate limiting for activity; however, it remains to be seen if there is an optimal configuration of Rp and Sp isomers that produce ASOs more active relative to the stereo-random parent or if the stereo-random parent is the optimal RNase H motif.

In this study, the 8-base gap ASOs (**A13**–**A16**) remain an outlying and interesting case. While the *T*_m_ follows the general trend of the 10-base gap (i.e. Rp>Sp), the *T*_m_, cell culture data and RNase H results are uncorrelated. Unlike the 10-base gap, the 8-base gap ASOs are nearly identical in cell culture, while the Sp isomer is more active against RNase H (Figure 4d). This again argues against RNase H being a rate limiting step with respect to activity, as the more RNaseH active Sp isomer is not more active in cell culture. Also, it seems likely these results are related to the shortened length, as the sequence does not differ significantly from the 10-base gap (i.e. **A11**/**A15** and **A12**/**A16**). Additionally, the cleavage patterns, as determined by the gels in Figure 4, do not adopt a different pattern from the 10-base gap ASOs, indicating that the preferred sites of cleavage are not significantly affected by shortening the gap. It remains to be seen if the results of these gap shortened compounds are *sui generis*, or if these results extend as a general pattern. One possibility, if this is indeed a general pattern, is that the conformational transmission is enhanced in the Rp isomer (because the cleavage site is closer to the RNA-like wings, resulting in a lower cleavage rate) over that of the Sp isomer; however, the potential structural reasons for this are unclear.

While initially it appeared to us, based on the *in vitro* assays, that ASOs with high Rp content was a positive attribute, the results from the *in vivo* studies challenged this assumption. Namely, the stereo-random isomer (**A6**) was more active than either the Rp isomer (**A7**) or the Sp isomer (**A8**), despite the fact that the Rp isomer was significantly more active *in vitro* than the Sp isomer. However, after comparing the amount of intact ASO in liver tissue (Figure 5b), it seems that some unidentified nuclease(s) is responsible for ASO metabolism and this nuclease(s) has a preference for the Rp isomer. After testing a series of nucleases (Supplementary Data, Figure S8), it was ascertained that some nucleases have a preference for the Sp isomer (e.g. S1) and others (e.g. micrococcal endonuclease) have a preference for the Rp isomer. Currently, the specific nuclease(s) responsible for rapid degradation of **A7** *in vivo* has yet to be identified.

The PS linkage is not limited in its use in RNase H mediated ASOs mechanisms. The PS linkage has been utilized for enhancing metabolic stability of immunomodulatory and other oligonucleotides which function through RISC pathways. It is conceivable that chirality of the PS linkage could impact the therapeutic properties of these oligonucleotides and the role that the PS moiety plays in their binding, activity and metabolism remains to be seen. However, controlling chirality of the PS linkage in the gap region of RNase H active gapmer ASOs provides no discernible benefits for therapeutic applications relative to the mixture of stereo-random PS ASOs. Furthermore, the added complexity and cost associated with the synthesis and characterization of chiral PS ASOs will likely limit their utility for broader applications.

## SUPPLEMENTARY DATA

Supplementary Data are available at NAR Online.

SUPPLEMENTARY DATA
