# Supplementary material for: Synthesis, biophysical properties and biological activity of second generation antisense oligonucleotides containing chiral phosphorothioate linkages
Source: Nucleic Acids Res. 2014 Nov 14;42(22):13456–68. doi: 10.1093/nar/gku1115 (PMC4267618; doi:10.1093/nar/gku1115)
Supplement: SUPPLEMENTARY DATA [file supp_42_22_13456__index.html]

Synthesis, biophysical properties and biological activity of second generation antisense oligonucleotides containing chiral phosphorothioate linkages — Synthesis, biophysical properties and biological activity of second generation antisense oligonucleotides containing chiral phosphorothioate linkages — SUPPLEMENTARY DATA 

# Synthesis, biophysical properties and biological activity of second generation antisense oligonucleotides containing chiral phosphorothioate linkages

## SUPPLEMENTARY DATA

**Files in this Data Supplement:**

- SUPPLEMENTARY DATA
