# Supplementary material for: Synthesis, biophysical properties and biological activity of second generation antisense oligonucleotides containing chiral phosphorothioate linkages
Source: Nucleic Acids Res. 2014 Nov 14;42(22):13456–68. doi: 10.1093/nar/gku1115 (PMC4267618; doi:10.1093/nar/gku1115)
Supplement: SUPPLEMENTARY DATA [file supp_gku1115_nar-01624-y-2014-File010.pdf]

Supplementary Information

Synthesis, Biophysical Properties and Biological Activity of Second Generation Antisense Oligonucleotides Containing Chiral Phosphorothioate Linkages

W. Brad Wan*, Michael T. Migawa, Guillermo Vasquez, Heather M. Murray, Josh G. Nichols, Hans Gaus, Andres Berdeja, Sam Lee, Christopher E. Hart, Walt F. Lima, Eric E. Swayze and Punit P. Seth

Isis Pharmaceuticals, Inc., 2855 Gazelle Ct, Carlsbad, CA 92010, USA

* To whom correspondence should be addressed. Tel: +1 760 931 9200; Fax: +1 760 603 2540; Email: bwan@isisph.com

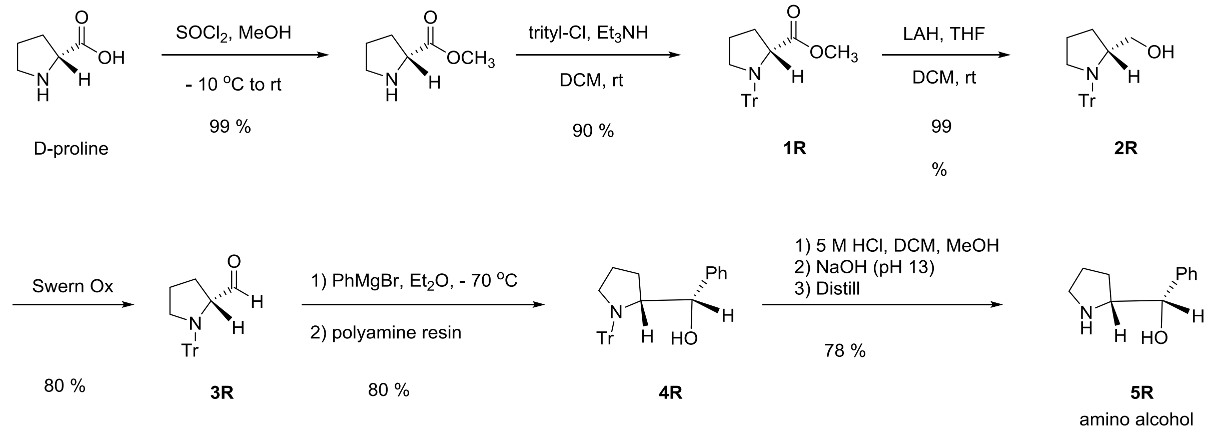

Supplementary Figure S1. Modified synthesis of enantiomerically pure amino alcohol **5R**. Trityl protected aldehyde **3R** was prepared from enantiomerically pure D-proline following a literature procedure (1).

Compound 4R. To a 3-neck, 5 L round bottom flask equipped with a thermocouple and nitrogen line was added compound **3R** (231 g, 0.676 mol), followed by anhydrous diethyl ether (2 L). The reaction was cooled to -78 °C using dry ice/ acetone, and phenyl magnesium bromide (3.0 M in diethyl ether, 450 mL, 1.35 mol, 2.0 eq) was added dropwise. After the addition was complete, the reaction continued to stir under nitrogen for 6 hours at -78 °C. The reaction was then quenched at -78 °C by adding, dropwise, a 900 ml solution mixture (2/1) of saturated aqueous NH_4Cl and NH_3 (28% in water). The reaction was then allowed to warm to room temperature, and the two layers were separated. The aqueous layer was extracted twice with EtOAc, and the combined organic phases were dried over anhydrous Na_2SO_4, filtered, and the solvent removed in vacuo. The resulting crude solid was dissolved in 500 mL MeOH/DCM (1:1 v/v) and treated with 100 grams of prepared resin* overnight. The resin was then removed by filtration and was washed with DCM. The filtrate was evaporated to dryness under reduced pressure (rotovap). Methanol (500 mL) was added and the product crystallized. The solids were collected by filtration and dried under high vacuum to give **4R** as a white solid (80 % yield).

***Prepared resin**. Tris-amine resin (100g, Aldrich, product number 472107) was treated with 1.0 M HCl (500mL), water (3 x 500mL), then MeOH (3x, 500mL).

Compound 5R. To a 2L round bottom flask with attached mechanical stirrer was placed compound **4R** (225 g, 0.536 mol) followed by 1:1 MeOH/DCM (300 mL) at room temperature. HCl (5.0 M aqueous solution, 400 mL) was added, and after 3 hours of vigorous stirring, the MeOH and DCM were evaporated, leaving the aqueous layer behind. The aqueous layer was extracted with 3 x 200 ml diethyl ether to remove the trityl byproduct. The aqueous phase was collected and cooled to 0 °C. The pH was adjusted to 12-13 using concentrated aqueous NaOH. The aqueous layer was then extracted with DCM (3 x 200mL), making sure to maintain pH = 12-13. The organic layers were then dried over Na_2SO_4 and then evaporated under reduced pressure to afford compound **5R** as an oil. The oil was purified by short path distillation under reduced pressure (~0.4 Torr, 145 °C external bath, 105-110 °C internal) to give 74 grams of **5R** (78% yield).

Compound **4L** and **5L** were prepared in an identical fashion from L-proline.

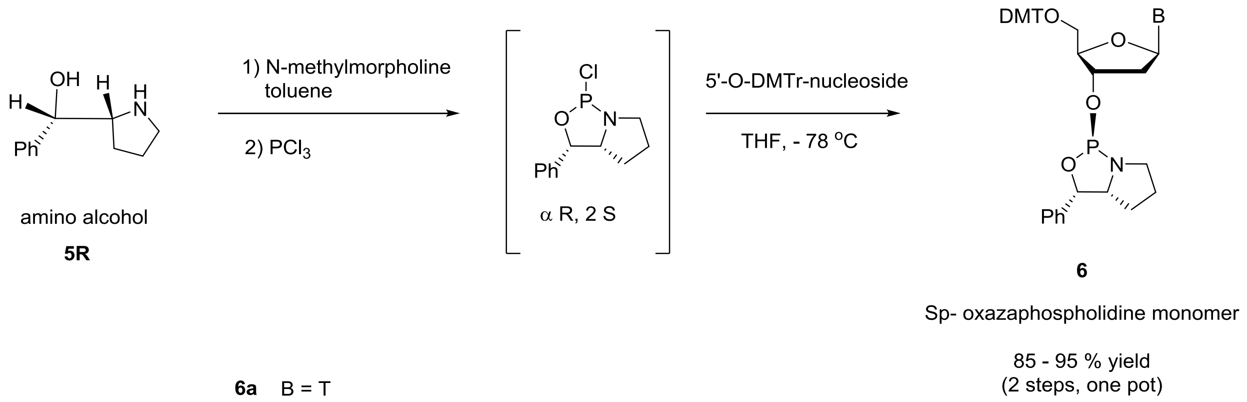

6a B = T
6b B = C^{Bz}
6c B = G^{iBu}
6d B = A^{Bz}

Supplementary Figure S2. One-Pot synthesis of OAP monomers. The synthesis of Sp-oxazaphospholidine thymidine (**6a**) is shown in Figure S2. All glassware and reagents were dried thoroughly prior to use. Amino alcohol **5R** (20 g, 0.112 mol) was dissolved in anhydrous toluene (80 ml) in a 250 ml round bottom flask with stirring under nitrogen. To this solution was added N-methylmorpholine (24.04 mL, 0.225 mol, 2.0 eq). A second 250 ml round bottom flask, equipped with magnetic stir bar, was charged with anhydrous toluene (150 ml) and phosphorus trichloride (9.34 mL, 0.107 mol, 0.95 eq) at -70 °C with stirring under nitrogen. The amino alcohol solution was transferred to the solution of PCl_3 via cannula over a period of 30 minutes. The reaction was allowed to warm to room temperature and was stirred for 40 minutes.

A third round bottom flask (1 liter) was equipped with magnetic stir bar and was charged with anhydrous THF (250 ml) , 5'-O-DMTr-thymidine (30.45g, 0.055 mol), and triethylamine **(**78 ml, 0.559 mol, 5.0 eq) with stirring under nitrogen at -70 °C. The solution of 2-chloro-oxazaphospholidine intermediate was added via cannula over a period of 30 minutes. The reaction was allowed to warm to room temperature and was stirred for 40 minutes. The reaction was cooled to – 20 °C and was quenched with saturated aqueous sodium bicarbonate solution (300 ml). The mixture was diluted with EtOAc (300 ml) and was transferred to a separatory funnel. The organics were collected, and were washed with saturated Na_2HCO_3 solution, H_2O, and brine (300 ml each). The combined organics were dried over Na_2SO_4, filtered, and evaporated to white foam under reduced pressure. The crude material was

dissolved in minimum amount of anhydrous THF and was passed through a plug of silica gel. The silica plug was flushed with anhydrous THF (300 mL), and the filtrate was concentrated give **6a** as a white, brittle foam (38.51 g, 93 % yield).

The same method, using identical quantities of reagents (on a molar basis) was used to prepare Sp-OAP monomers **6b** – **6d** from appropriately protected nucleosides. The Rp OAP monomers (**7a -7d**) were also prepared using this method, starting from amino alcohol **5L.** Characterization of all monomers (^{1}H NMR and ^{31}P NMR) was consistent with that reported by Wada *et al*. (2).

Compound	Yield
6a	93 %
6b	86 %
6c	91 %
6d	88 %
7a	95 %
7b	95 %
7c	88 %
7d	89 %

Product, 5' → 3' [a,b]	Ratio of Stereoselectivity [c]	Product, 5' → 3' [a]	Ratio of Stereoselectivity [c]
DMT-AA-OH	44 : 56	DMT-ATTT^{m}CAGT^{m}CAGT^{m}CA-OH	41 : 59
DMT-A$_{Rp}$A-OH	4 : 96		
DMT-AA$_{Rp}$A-OH	42 : 58	DMT-A$_{Rp}$TTT^{m}CAGT^{m}CAGT^{m}CA-OH	2 : 98
DMT-^{m}CA$_{Rp}$A-OH	41 : 59	DMT-^{m}C$_{Rp}$TTT^{m}CAGT^{m}CAGT^{m}CA-OH	2: 98
DMT-TA$_{Rp}$A-OH	44 : 56	DMT-T$_{Rp}$TTT^{m}CAGT^{m}CAGT^{m}CA-OH	2 : 98
DMT-GA$_{Rp}$A-OH	47 : 53	DMT-G$_{Rp}$TTT^{m}CAGT^{m}CAGT^{m}CA-OH	2 : 98
DMT-AA-OH	44 : 56	DMT-ATTT^{m}CAGT^{m}CAGT^{m}CA-OH	41 : 59
DMT-A$_{Sp}$A-OH	96 : 4		
DMT-AA$_{Sp}$A-OH	40 : 60	DMT-A$_{Sp}$TTT^{m}CAGT^{m}CAGT^{m}CA-OH	98 : 2
DMT-^{m}CA$_{Sp}$A-OH	41 : 59	DMT-mC$_{Sp}$TTT^{m}CAGT^{m}CAGT^{m}CA-OH	98 : 2
DMT-TA$_{Sp}$A-OH	45 : 55	DMT-T$_{Sp}$TTT^{m}CAGT^{m}CAGT^{m}CA-OH	97 : 3
DMT-GA$_{Sp}$A-OH	48 : 52	DMT-G$_{Sp}$TTT^{m}CAGT^{m}CAGT^{m}CA-OH	97 : 3

[a] Each nucleoside followed by a subscript "Sp" indicates a 3'-Sp linkage, and each nucleoside followed by a subscript "Rp" indicates a 3'-Rp linkage. All other linkages are stereo-random PS.
[b] Dimers and trimers were prepared on polystyrene support pre-loaded with 2'-deoxyadenosine at 200 µmol/gram
[c] Stereoselectivity of 5'-terminal coupling was determined by analytical HPLC (UV Area)

Supplementary Figure S3: Evaluation of the stereospecific couplings method. To further evaluate the stereoselectivity of the coupling, an OAP monomer was coupled to a polystyrene resin that was pre-loaded with deoxy adenosine at 200 µmol/gram. The resulting dimer was cleaved from the resin (DMT on), and the stereospecificity was determined by analytical HPLC. A third coupling was performed under racemic conditions in order to determine whether or not the stereo-selectivity of a given coupling is independent of the stereochemistry of the preceding base. The results in table **S3** reaffirms that OAP monomers can provide highly enriched stereo-selective couplings (~ 97 % pure), and confirms that the stereoselectivity of each coupling is independent of the stereochemistry of the preceding nucleoside.

Product, 5' → 3' [a]	Activator [b]	Capping reagent	Sulfurizing reagent [c]	Ratio of Stereoselectivity [d]	Unreacted 14-mer
DMT-T_{Sp}TTT^{m}CAGT^{m}CAGT^{m}CA-OH	CMPT	TF-imid	PADS	98 : 2	12 %
DMT-T_{Sp}TTT^{m}CAGT^{m}CAGT^{m}CA-OH	CMPT	TF-imid	Beaucage	98 : 2	11%
DMT-T_{Sp}TTT^{m}CAGT^{m}CAGT^{m}CA-OH	CMPT	TF-imid	XH	98 : 2	12 %
DMT-T_{Rp}TTT^{m}CAGT^{m}CAGT^{m}CA-OH	CMPT	TF-imid	PADS	2 : 98	10 %
DMT-T_{Sp}TTT^{m}CAGT^{m}CAGT^{m}CA-OH	DCI	TF-imid	PADS	98 : 2	11 %
DMT-T_{Sp}TTT^{m}CAGT^{m}CAGT^{m}CA-OH	DCI	Ac_2O	PADS	98 : 2	10 %
DMT-T_{Sp}TTT^{m}CAGT^{m}CAGT^{m}CA-OH	ETT	TF-imid	PADS	98 : 2	14 %
DMT-T_{Sp}TTT^{m}CAGT^{m}CAGT^{m}CA-OH	Tetrazole	TF-imid	PADS	98 : 2	32 %

[a] Each nucleoside followed by a subscript “Sp" indicates a 3’-Sp linkage, and each nucleoside followed by a subscript “Rp" indicates a 3’-Rp linkage. All other linkages are stereo-random PS.
[b] CMPT = N-(cyamnomethyl)pyrrolidinium triflate
[c] XH = xanthane hydride, PADS = phenylacetyl disulfide
[d] Stereoselectivity of 5’-terminal coupling was determined by analytical HPLC (UV Area)

Supplementary Figure S4: Varying coupling conditions and reagents did not affect the stereoselectivity of the OAP couplings. During the optimization process, several different conditions/reagents were investigated. We found that the method was tolerant to a variety of reagents at each step. Choice of activator (CMPT, DCI, tetrazole, ETT), choice of sulfurization reagent (Beaucage reagent, DTD, PADS, XH), and/or capping reagent (Tf-imidazole, Tf-benzotriazole, or Ac_2O) (3) did not affect the stereochemical outcome of the coupling reaction. Figure **S4** summarizes some of the conditions that were tried. OAP was prepared at 0.1 M in 1:1 ACN/toluene. Activator was added in 10-fold excess relative to OAP, and coupling time was 5 minutes. Although Tf-imidazole is used commonly for OAP chemistry, we found the reagent to be extremely hydroscopic and to have an insufficient shelf life. We found that acetic anhydride to be more compatible with our automated synthesizer, however cleavage of the chiral auxiliary was significantly slower (48 hours, concentrated NH_4OH, 55 °C). The coupling efficiency was fairly consistent regardless of conditions used. The conditions were later optimized to consistently give an estimated 93 % coupling efficiency by increasing OAP concentration to 0.2 M, and utilizing a “double coupling” (i.e. two applications of OAP and activator, 6 minutes each), as reflected in the Materials and Methods section.

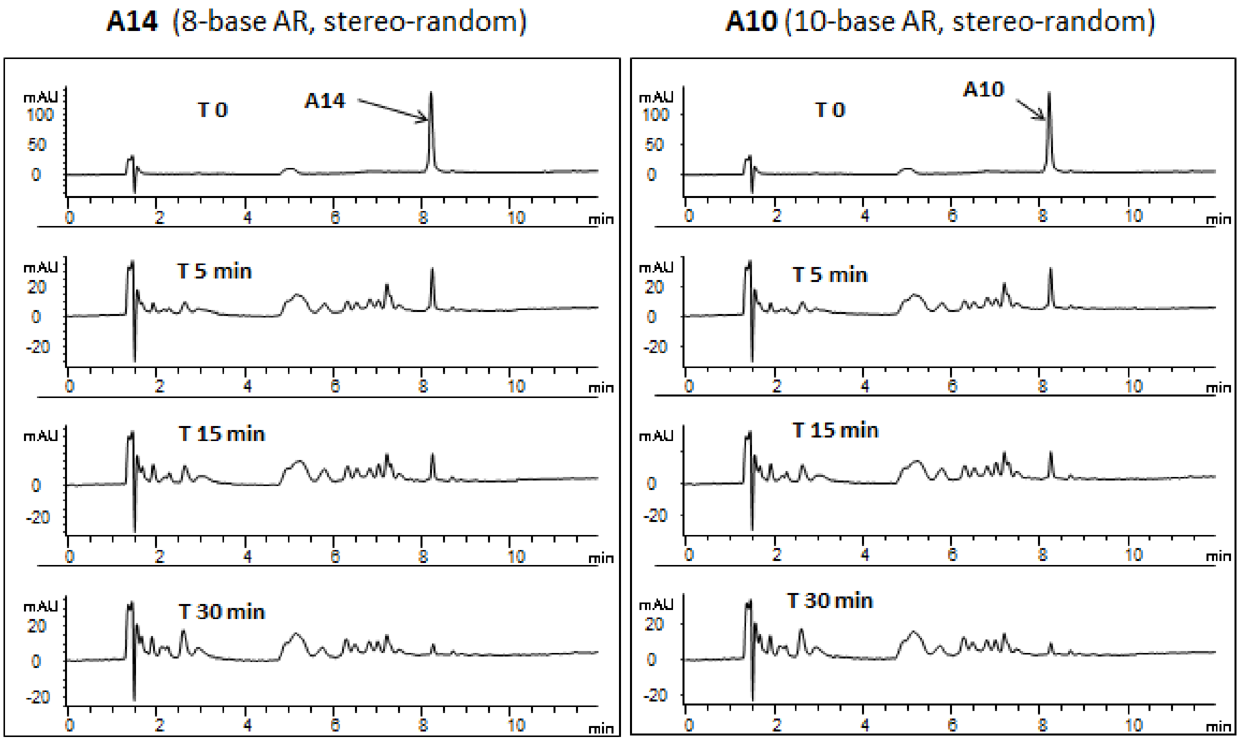

Supplementary Figure S5: S1endonuclease digest of racemic gap AR ASOs **A14** and **A10**. Nuclease S1 *Aspergillus oryzae* was purchased from Sigma (Sigma; N5661-50KU; 223196 units/mL). ASO digestion reaction was performed in 60 mM NaOAc, 1 mM $ZnCl_2$, pH 4.6 at room temperature, 112 units/mL. ASOs solutions were prepared at 5 µM ASO concentration, 100 µl reaction volume. 20 µl of reaction volume was quenched with 20 µL of 12.5 mM EDTA solution and enzyme inactivated through heating solution to 80 °C for 5 minutes. Samples were run on LC-MS instrument over a time course; 35 µL injection. The digestion profile showed that both of the racemic parent ASOs compounds (**A14** and **A10**) were completely digested within 30 minutes under the reaction conditions described above.

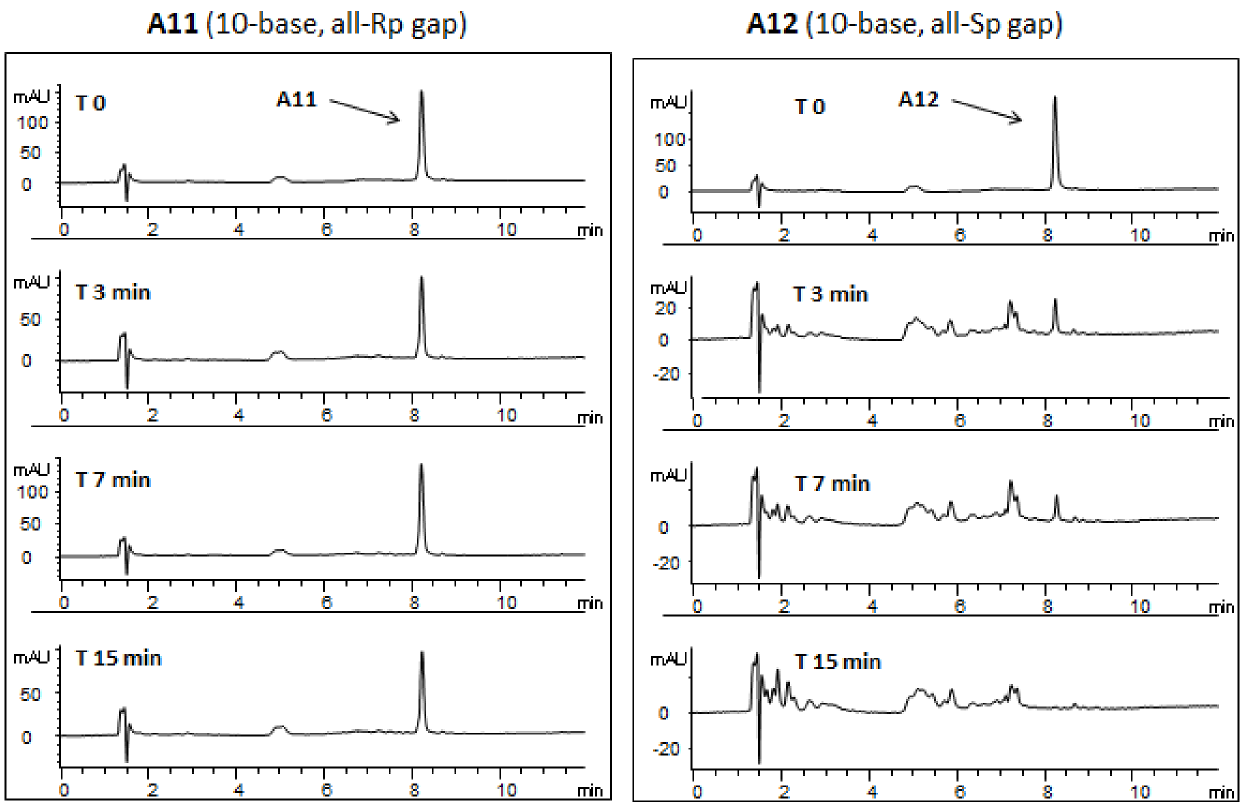

Supplementary Figure S6: S1 endonuclease digest of 10-base P-chiral gap AR ASOs **A11** and **A12**. For experimental conditions, see Figure S5. The digestion profile showed that the 10-base all-Sp-gap ASO (**A12**) was rapidly degraded by S1 endonuclease while the Rp-gap ASO (**A11**) was found to be stable.

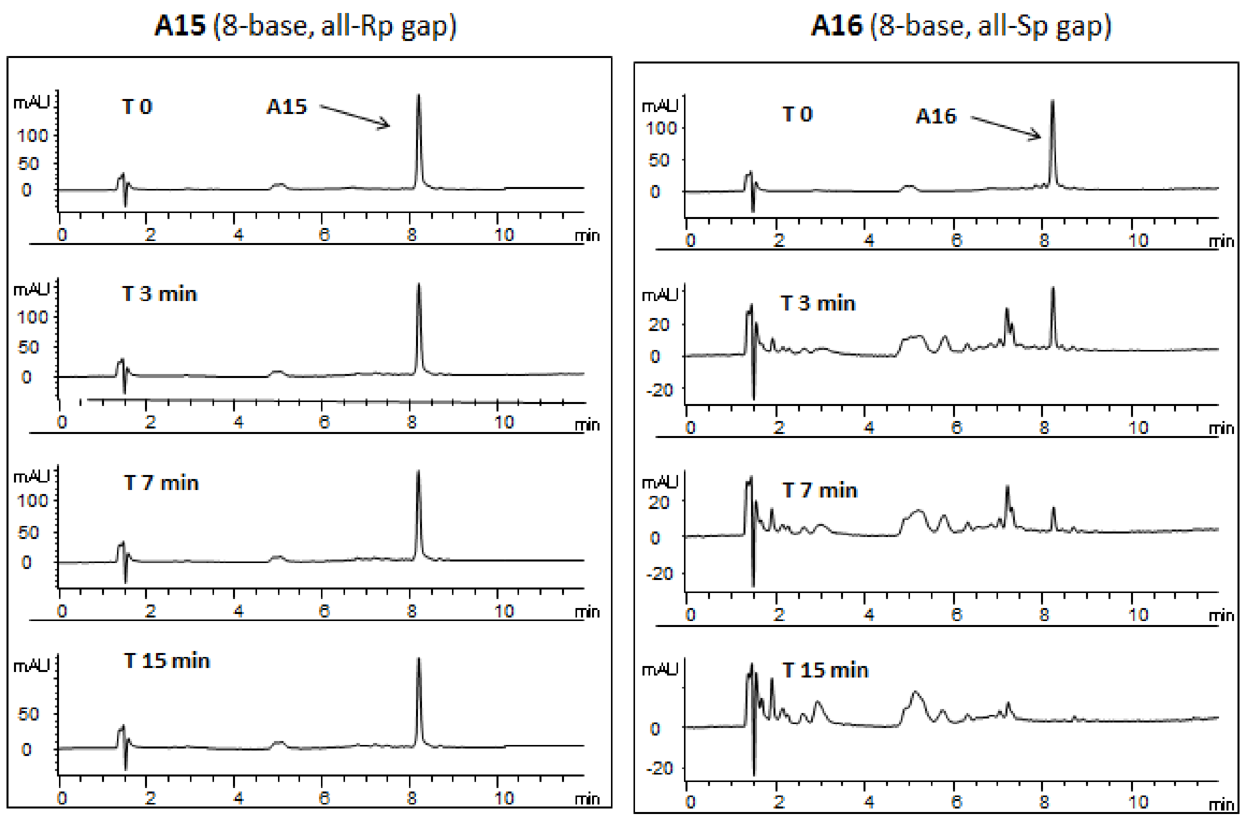

Supplementary Figure S7: S1 endonuclease digest of 8-base P-chiral gap AR ASOs **A15** and **A16**. For experimental conditions, see Figure S5. The digestion profile showed that the 8-base all-Sp gap ASO (**A16**) was rapidly degraded by S1 endonuclease while the Rp-gap ASO (**A15**) was found to be stable.

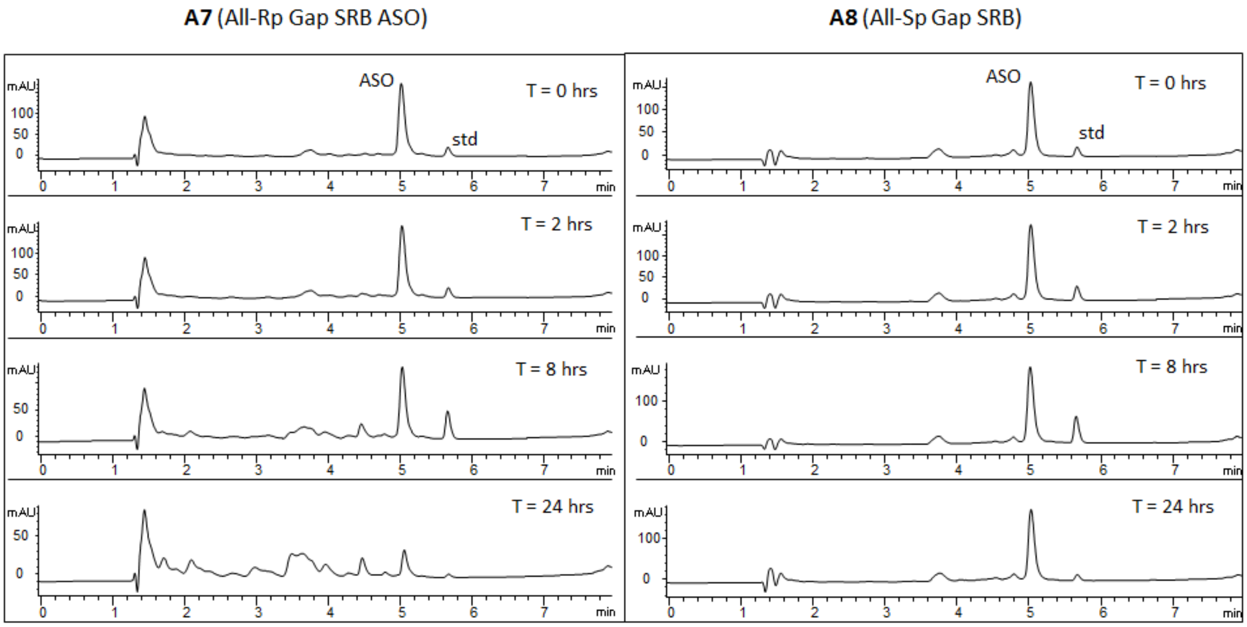

Supplementary Figure S8: Micrococcal endonuclease digest of P-chiral gap SRB ASOs **A7** and **A8**. Micrococcal endonuclease from *Staphylococcus aurenus* was purchased from Sigma (Sigma; N3755-50UN). ASO digestion reaction was performed according to a literature procedure (4). Briefly, the enzyme was prepared and stored in 20 mM tris-HCl at pH 7.6, 50 mM NaCl, and 50 % glycerol (v/v); final concentration of enzyme was 458 units/mL. Reaction buffer was 10 µM Tris-HCl (H = 8) and 1 mM $CaCl_2$. ASO solutions were prepared at 10 µM ASO concentration in reaction buffer, 100 µl reaction volume, 5 units of enzyme per reaction. Samples were incubated at 37 °C for 0, 2, 8, and 24 hours. Reactions were quenched with 100 µL of a quenching buffer (8 M urea, 50 mM EDTA, and 0.5 mM of an internal standard ASO) and the enzyme inactivated through heating solution to 80 °C for 5 minutes. Samples were run on LC-MS instrument over a time course; 35 µL injection. The digestion profile showed that the all-Rp gap ASO (**A7**) was rapidly degraded by micrococcal endonuclease while the all Sp-gap ASO (**A8**) was found to be stable.

A similar assay was performed using svPDE, porcine DNA Polymerase I, DNase II, human APE-1, and mung bean endonuclease. Stability tests of **A7** and **A8** were performed in mouse serum and mouse hepatocyte homogenate. In all cases, both **A7** and **A8** were found to be stable (t ½ >> 24 hours), and there was no significant difference was observed between the all-Sp and the all-Rp gap compounds.

	Molecular weight (LCMS)			Molecular weight (LCMS)	
ASO	Calculated	Found	ASO	Calculated	Found
**A2**	4647.0	4646.41	**A31**	5370.6	5369.4
**A3**	4647.0	4645.8	**A32**	5370.6	5369.1
**A4**	4647.0	4646.1	**A33**	5370.6	5369.4
**A6**	5370.6	5369.7	**A34**	5370.6	5369.4
**A7**	5370.6	5369.1	**A35**	5370.6	5369.1
**A8**	5370.6	5369.1	**A36**	5370.6	5369.4
**A10**	5457.6	5456.7	**A37**	5370.6	5369.4
**A11**	5457.6	5456.4	**A38**	5370.6	5369.4
**A12**	5457.6	5456.4	**A39**	5370.6	5369.4
**A14**	5669.8	5668.5	**A40**	5370.6	5369.4
**A15**	5669.8	5668.5	**A41**	5370.6	5369.4
**A16**	5669.8	5668.5	**A42**	5370.6	5369.1
**A18**	3722.6	3721.5	**A43**	5370.6	5369.4
**A19**	3754.7	3753.6	**A44**	5370.6	5369.4
**A20**	3786.9	3786.0	**A45**	5370.6	5369.4
**A21**	3722.6	3721.5	**A46**	5370.6	5369.4
**A22**	3754.7	3753.6	**A47**	5370.6	5369.4
**A23**	3786.9	3785.7	**A48**	5370.6	5369.1
**A24**	3786.9	3785.7	**A49**	5370.6	5369.4
**A25**	3786.9	3785.7	**A50**	5370.6	5369.1
**A26**	3786.9	3786.0	**A51**	5370.6	5369.1
**A27**	5370.6	5369.4	**A52**	5370.6	5369.4
**A28**	5370.6	5369.4	**A53**	5370.6	5369.1
**A29**	5370.6	5369.4	**A54**	5370.6	5369.4
**A30**	5370.6	5369.1	**A55**	5370.6	5369.1

Supplementary Figure S9: Mass spectral data for ASOs presented in this report

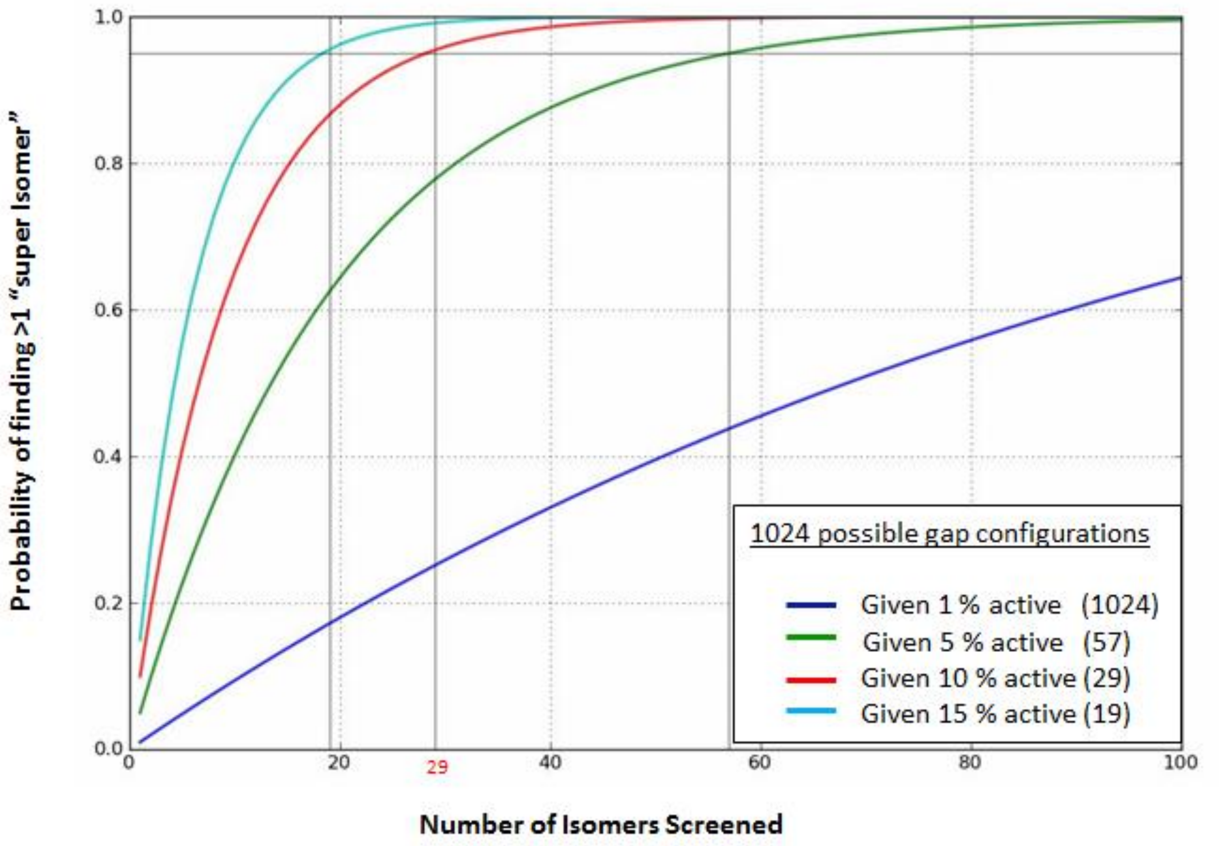

Supplementary Figure S10:

The probability of finding a "super isomer" (defined as an isomer that is 5x more potent than the stereo-random parent ASO) depends on the number of isomers screened, and the number of "super-isomers" present in the population. Considering a 10-base gapmer motif, there are 1024 possible gap configurations. The number in parenthesis is the number of isomers necessary to be screened to achieve 95 % confidence of finding a "super isomer" based on the assumption that either 1, 5, 10, or 15 % of the total isomer population is a super isomer. 29 isomers were tested in this study.

References:

1) Natsuhisa Oka, T.W., and Kazuhiko Saigo. (2003) An Oxazaphospholidine Approach for the Stereocontrolled Synthesis of Oligonucleoside Phosphorothioates. *J. Am. Chem. Soc.*, **124,** 8307-8317.
2) Oka, N., Yamamoto, M., Sato, T. and Wada, T. (2008) Solid-Phase Synthesis of Stereoregular Oligodeoxyribonucleoside Phosphorothioates Using Bicyclic Oxazaphospholidine Derivatives as Monomer Units. *J. Am. Chem. Soc..,* **130**, 16031-16037
3) Katritzky, A.R., Yang, B. and Semenzin, D. (1997) (Trifluoroacetyl)benzotriazole: A Convenient Trifluoroacetylating Reagent. *J. Org. Chem*, **62,** 726-728.
4) Nichols, N.M. (2011) Endonucleases. Current Protocols Mol Bio, Chapter 3, Unit 3.12
